# Diabetes Diminishes the Portal-Systemic Collateral Vascular Response to Vasopressin via Vasopressin Receptor and G_α_ Proteins Regulations in Cirrhotic Rats

**DOI:** 10.1371/journal.pone.0067703

**Published:** 2013-07-09

**Authors:** Jing-Yi Lee, Teh-Ia Huo, Sun-Sang Wang, Hui-Chun Huang, Fa-Yauh Lee, Han-Chieh Lin, Chiao-Lin Chuang, Shou-Dong Lee

**Affiliations:** 1 Institute of Pharmacology, National Yang-Ming University, Taipei, Taiwan; 2 Faculty of Medicine, National Yang-Ming University, Taipei, Taiwan; 3 Divisions of General Medicine, Taipei Veterans General Hospital, Taipei, Taiwan; 4 Gastroenterology, Department of Medicine, Taipei Veterans General Hospital, Taipei, Taiwan; 5 Department of Medical Affair and Planning, Taipei Veterans General Hospital, Taipei, Taiwan; 6 Division of Gastroenterology, Department of Medicine, Cheng Hsin General Hospital, Taipei, Taiwan; University of Colorado, United States of America

## Abstract

Liver cirrhosis may lead to portal-systemic collateral formation and bleeding. The hemostatic effect is influenced by the response of collateral vessels to vasoconstrictors. Diabetes and glucose also influence vasoresponsiveness, but their net effect on collaterals remains unexplored. This study investigated the impact of diabetes or glucose application on portal-systemic collateral vasoresponsiveness to arginine vasopressin (AVP) in cirrhosis. Spraque-Dawley rats with bile duct ligation (BDL)-induced cirrhosis received vehicle (citrate buffer) or streptozotocin (diabetic, BDL/STZ). The *in situ* collateral perfusion was done after hemodynamic measurements: Both were perfused with Krebs solution, D-glucose, or D-glucose and NaF, with additional OPC-31260 for the BDL/STZ group. Splenorenal shunt vasopressin receptors and G_α_ proteins mRNA expressions were evaluated. The survival rate of cirrhotic rats was decreased by STZ injection. The collateral perfusion pressure changes to AVP were lower in STZ-injected groups, which were reversed by OPC-31260 (a V_2_R antagonist) and overcome by NaF (a G protein activator). The splenorenal shunt V_2_R mRNA expression was increased while G_α_ proteins mRNA expressions were decreased in BDL/STZ rats compared to BDL rats. The G_αq_ and G_α11_ mRNA expressions also correlated with the maximal perfusion pressure changes to AVP. Diabetes diminished the portal-systemic collateral vascular response to AVP in rats with BDL-induced cirrhosis, probably via V_2_ receptor up-regulation and G_α_ proteins down-regulation.

## Introduction

Liver cirrhosis is characterized by increased intrahepatic resistance and splanchnic hyperemia with elevated portal pressure, the so-called portal hypertension [1]. As portal pressure increases, portal-systemic collaterals develop to divert portal blood flow to the systemic circulation, among which gastroesophageal varices are the most prominent [2]. Vascular endothelial growth factor, a vascular endothelium-derived factor mediating vasodilation and angiogenesis, also participates in the development of portal hypertension and varices [3], [4]. Variceal hemorrhage is the most dreadful and challenging complication [5]. The current treatment strategy aims to reduce portal inflow, portal pressure and/or constrict collateral vasculature. Therefore, factors that alter collateral vascular contractility may contribute to the control of variceal hemorrhage.

Arginine vasopressin (AVP) and its long-acting analogue terlipressin have been used to control variceal bleeding and hepatorenal syndrome [6]. Recently, AVP has been shown to directly constrict portal-systemic collaterals of portal hypertensive and bile duct-ligated (BDL) cirrhotic rats [7], [8]. The vascular effects of AVP begin with its binding to G-protein-coupled V_1a_ receptor. Subsequently, the activation of G-protein α-subunits like G_αq_ and G_α11_ recruits protein kinase C and phospholipase C. They release Ca^2+^ from intracellular stores and induce vasoconstriction [9].

The liver regulates blood glucose levels by gluconeogenesis and glycogenolysis, which are essential in carbohydrate metabolism. In cirrhosis, impaired insulin-mediated glucose uptake contributes to profound and sustained hyperglycemia [10]. Previous analysis showed that up to 96% of cirrhotic patients were glucose intolerant and 30% might develop clinical diabetes [11], [12]. Long-standing diabetes may lead to structural and functional vascular anomalies [13]. Hyperglycemia may cause divergent vascular actions in different situations. In previous investigations, hyperglycemia impaired the endothelium-dependent relaxation of rat isolated mesenteric resistance arteries [14] and acetylcholine-induced vasodilation of renal arterioles [15]. *In vivo* studies indicated that hyperglycemia also significantly suppressed endothelium-mediated vasodilation of rats with streptozotocin (STZ)-induced diabetes [16]. On the other hand, abolished Ca^2+^ transients in response to physiological agonists have been reported in vascular smooth muscle cells of diabetic animals [17]. In diabetic rats, aortic smooth muscle and portal vein contractile response to vasoactivators were abolished [18]. In addition, renal perfusion pressure responses to AVP were markedly blunted in diabetic rats [19].

**Figure 1:**
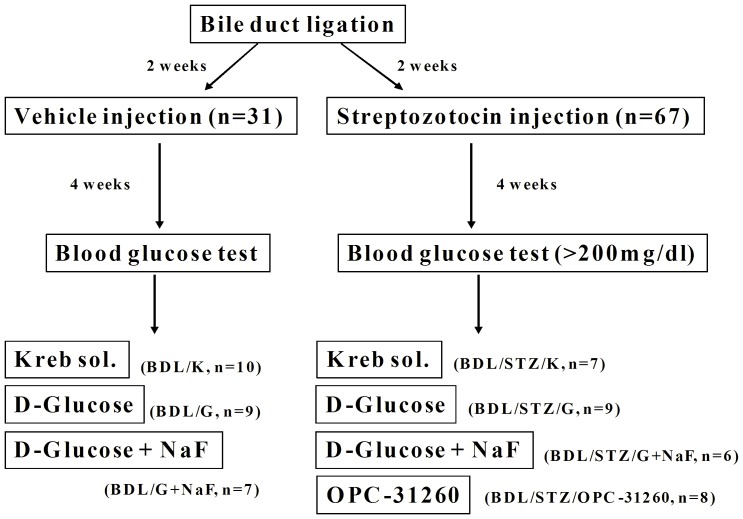
Study design.

Hyperglycemia and glucose application alter vascular reactivity in different vascular beds, but the corresponding influences on the portal-systemic collateral vascular bed of cirrhosis have not yet been surveyed. We hypothesized that diabetes and/or glucose application may elicit changes of collateral vasoresponsiveness to AVP in cirrhotic rats. Furthermore, the results may provide the information capable of improving the treatment efficacy of vasoconstrictors in cirrhotic and diabetic patients with variceal hemorrhage.

## Materials and Methods

### Animal model: common bile-duct ligation (BDL)

Male Sprague-Dawley rats (240–270 g) received BDL to induce liver cirrhosis [20]. Under ketamine anesthesia (100 mg/kg, intramuscularly), the common bile duct was exposed and catheterized by a PE-10 catheter, followed by 3–0 silk ligation. Then 10% formalin (∼100 μl/100 g body weight) was slowly injected into the biliary tree to prevent the subsequent dilatation of the ligated residual bile duct. The common bile duct was cut between the ligatures after the PE-10 catheter was removed and the ligatures were tightened. Secondary biliary cirrhosis was noted since five weeks after ligation. To avoid coagulation defects, BDL rats were given vitamin K injection (50 μg/kg intramuscularly) weekly.

This study was approved by Taipei Veterans General Hospital Animal Committee (IUACU no. 99–123) and was conducted according to the principles of laboratory animal care [Guide for the Care and Use of Laboratory Animals (1985), DHEW Publication no. (NIH) 85–23: Office of Science and Health Reports, DRR/NIH, Bethesda, MD, U.S.A.].

### Experimental model of diabetes

Diabetes was induced by intraperitoneal injection of STZ (60 mg/kg in citrate buffer) 2 weeks after BDL as previously described [21]. Blood samples were withdrawn from tail veins and blood glucose levels were assessed before hemodynamic measurement. Rats with blood glucose levels higher than 200 mg/dl were considered diabetic and eligible for the experiments. Rats with blood glucose levels between 100 and 200 mg/dl received an oral glucose tolerance test (OGTT): Blood glucose levels were measured 120 min after the D-glucose load (2 g/Kg). Rats with glucose levels higher than 200 mg/dl after OGTT were also considered diabetic.

### Systemic and portal hemodynamic measurements

The right femoral artery of BDL rats was cannulated with a PE-50 catheter connected to a Spectramed DTX transducer (Spectramed Inc., Oxnard, CA, USA). The abdomen was then opened with a mid-line incision and the mesenteric vein was cannulated with an 18-gauge Teflon cannula connected to a Spectramed DTX transducer. Continuous recordings of mean arterial pressure (MAP), heart rate (HR) and portal pressure (PP) were performed on a multi-channel recorder (model RS 3400, Gould Inc., Cupertino, CA, USA). The external zero reference was at the level of the right atrium.

### In situ perfusion of portal-systemic collaterals

The *in situ* perfusion system was performed as previously described [7], [8]. Both jugular veins were cannulated with 16-gauge Teflon cannulas to ensure an adequate outflow without any resistance. Heparin (200 u/100 g) was injected through one of the cannulas. The 18-gauge Teflon cannula inserted into the distal mesenteric vein served as the perfusate inlet. To exclude the liver from perfusion, the second loose ligature around the portal vein was tightened. The animal was then transferred into a warm chamber (37±0.5°C). An open circuit perfusion was then started with Krebs solution (composition in mM: NaCl, 118; KCl, 4.7; KH_2_PO_4_, 1.2; MgSO_4_, 1.2; CaCl_2_, 2.5; NaHCO_3_, 25; dextrose 11.0; pH 7.4, 37±0.5°C) containing 3% wt/vol albumin (factor V bovine serum albumin, Sigma, St. Louis, MO, USA) by a roller pump (model 505S, Watson-Marlow Limited, Falmouth, Cornwall, UK). The perfusate was equilibrated and oxygenated with carbogen gas (95% O_2_-5% CO_2_) by a silastic membrane lung. Pneumothorax was created by opening slits through the diaphragm to increase resistance in pulmonary arteries and prevent the perfusate from entering the left heart chambers. A Spectramed DTX transducer attached to the Gould model RS 3400 recorder was connected to continuously monitor and record continuously the pressure in the portal-systemic collateral vascular bed. All the experiments were performed 25 min after starting perfusion at a constant rate of 12 ml/min. In each individual preparation, the contracting capability of the portal-systemic collaterals was challenged with a 125-mM potassium chloride solution at the end of the experiments.

### Ribonucleic acid isolation and real-time PCR analysis

Splenorenal shunt, the most prominent intra-abdominal portal-systemic collateral was dissected and immediately stored in liquid nitrogen after the *in situ* perfusion experiments. Total RNA was extracted using the SV Total RNA Isolation System (Promega, USA). Total RNA (1 μg) was reverse-transcribed to cDNA with ImProm-II Reverse Transcriptase (Promega, Wisconsin, USA). Quantitative RT-PCR was performed by a LightCycler (LightCycler 480, Roche Diagnostics, Mannheim, Germany) and a standard LightCycler amplification cycle protocol was established.

The amplification cycle began with a denaturation program for 10 min at 95°C. cDNA was then amplified by 40 cycles of the time profiles as: 15 s at 95°C (denaturation), 30 s at 58°C for vasopressin receptors (V_1a_R, V_2_R), G proteins (G_αq_, G_α11_, G_αs_, G_αi_) and β-actin (annealing), and 10 s at 72°C (elongation). The melting curve program was performed for 0 s at 95°C, 15 s at 57°C and a linear temperature transition at 0.05°C·s^−1^ from 57°C to 95°C with continuous fluorescence acquisition. The final segment consisted of a cooling program to 40°C. The products were standardized with a housekeeping gene, β-actin, from the same RNA samples. Quantitative analysis was measured with LightCycler analysis software (Roche Diagnostics, Mannheim, Germany). Primer sequences of the target and housekeeping genes are listed in Table 1.

**Table 1:** Primers of target and housekeeping genes used for RT-PCR.

Names	Sense	Antisense	NCBI accession number
V_1a_R	F:5-CGCACTGTGAAGATGACCTTT-3	R:5-TGGAAGGGTTTTCTGAATCG-3	NM053019.2
V_2_R	F:5-ACCCTTCTTCCTCGTGCAG-3	R:5-AGCAGCATGAGCAACACAAA-3	NM019136.1
Gαq	F:5-GCACAATTGGTTCGAGAGGT-3	R:5-GATAGGAAGGGTCAGCCACA-3	NM031036
Gα11	F:5-CCGTTTGACCTGGAGAACAT-3	R:5-TCACAGACGAGTGCTGGAAC-3	NM031033
Gαs	F:5-CGTGCCAAACTTTGACTTCC-3	R:5-TGGCAGTCACATCATTGAAGC-3	NM021845
Gαi	F:5-TACAGCAACACCATCCAGTC-3	R:5-AAGTGGGTTTCTACGATGCC-3	NM017327
β-actin	F:5-CGCCCTAGGCACCAGGGTG-3	R:5-GCTGGGGTGTTGAAGGTCTCAAA-3	

### Experimental design (Figure 1)

Two weeks after BDL, 98 rats were randomly allocated to receive intraperitoneal injection of vehicle (cirrhotic control, BDL, n = 31) or STZ 60 mg/kg (cirrhotic and diabetic, BDL/STZ, n = 67). On the 43^rd^ day after BDL, the MAP, PP and HR were measured. Collateral vascular responsiveness to AVP (10^−10^–10^−7^ M) was evaluated with the following perfusions: BDL groups were perfused with (1) Krebs solution (BDL/K), (2) 45 mM D-glucose (BDL/G), or (3) 45 mM D-glucose and 10 mM sodium fluoride (NaF, a G protein activator; BDL/G+NaF); BDL/STZ groups were perfused with (4) Krebs solution (BDL/STZ/K), (5) 45 mM D-glucose (BDL/STZ/G), (6) 45 mM D-glucose and 10 mM NaF (BDL/STZ/G+NaF), or (7) 1 µM OPC-31260 (a selective vasopressin V_2_ receptor antagonist; BDL/STZ/OPC-31260). After the perfusion experiments, the splenorenal shunts of BDL and BDL/STZ rats perfused with Krebs solution were isolated and dissected for real-time PCR analysis.

### Drugs

AVP, streptozotocin, NaF, OPC-31260 and the reagents for preparing Krebs solution were purchased from Sigma (Sigma Chemical Co., St. Louis, MO, USA). All of the solutions were freshly prepared on the days of the experiments.

### Data Analysis

The results were expressed as mean±SDM. Changes in perfusion pressure (ΔmmHg) from baseline were calculated for each concentration in each rat. Statistical analyses were performed using the independent Student's *t*-test or one-way ANOVA followed by Bonferroni post-hoc test as appropriate. The survival rates of the control and diabetic groups were analyzed by log-rank test (Mantel-Cox). The concentration of vasopressin exhibiting 50% of the maximal response (EC50) in each preparation was calculated from the sigmoid logistic curves and expressed as nM. The results were considered statistically significant at a two-tailed P value less than 0.05. Simple linear regression was applied to analyze the correlation of splenorenal shunt G_α_ protein mRNA expressions and the maximal perfusion pressure changes to AVP.

## Results

### Survival rates of vehicle- (cirrhotic group, BDL) and STZ-injected (cirrhotic and diabetic group, BDL/STZ) BDL rats

On the 43^nd^ day after BDL, the survival rates of BDL and BDL/STZ groups were analyzed by log-rank test. The survival rate of the BDL group (87.1%) was significantly higher than that of the BDL/STZ group (49.3%, *P* = 0.0003).

### Baseline glucose and hemodynamic data

Only BDL/STZ rats with blood glucose levels in excess of 200 mg/dl were considered diabetic. The blood glucose level of vehicle-injected BDL rats was below 100 mg/dl. Compared to the BDL group, the BDL/STZ group had significantly lower baseline body weight (BW), MAP and HR (Table 2). There were no significant differences in glucose, BW, MAP, PP, and HR between BDL rats perfused with Krebs solution (BDL/K, n = 10), 45 mM D-glucose (BDL/G, n = 9), or 45 mM D-glucose and NaF (BDL/G+NaF, n = 7). The parameters were also similar among BDL/STZ rats perfused with Krebs solution (BDL/STZ/K, n = 7), 45 mM D-glucose (BDL/STZ/G, n = 9), 45 mM D-glucose and NaF (BDL/STZ/G+NaF, n = 6), or OPC-31260 (BDL/STZ/OPC-31260, n = 8) (*P*>0.05).

**Table 2:** Body weight, serum glucose concentration and hemodynamics in BDL rats with saline or STZ injection.

	n	BW (g)	glucose (mg·dl^−1^)	MAP (mmHg)	PP (mmHg)	HR (beats·min^−1^)
BDL	26	341±44	99.5±28.0	96.8±12.4	17.2±3.2	299±52
BDL/STZ	30	285±53†	390.3±109.4†	87.1±16.8*	16.6±2.6	263±48*

BW: body weight; MAP: mean arterial pressure; PP: portal pressure; HR: heart rate; BDL: bile duct ligation; STZ: streptozotocin.

* : *P*<0.05 vs. BDL rat; †: *P*<0.01 vs. BDL rat.

### Concentration-response relationships to AVP

#### a. Vehicle- vs. STZ-injected BDL rats with Krebs solution or D-glucose perfusion

**Figure 2:**
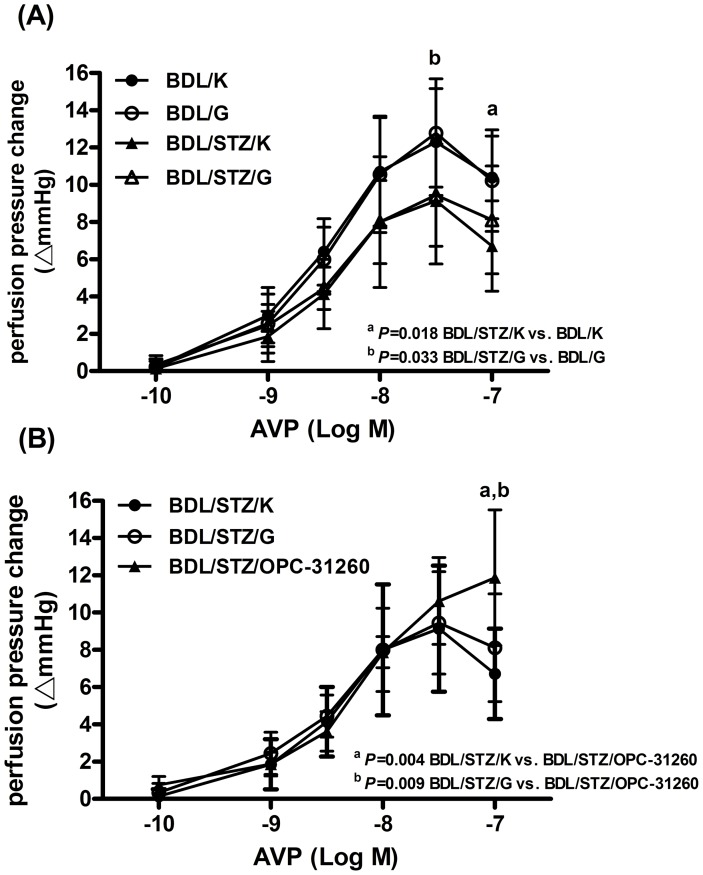
Concentration-response curves to AVP in portal-systemic collateral vascular beds. (A) Concentration-response curves of BDL and BDL/STZ rats. BDL: vehicle injection; BDL/STZ: STZ injection; K: Krebs solution; G: D-glucose, expressed as absolute increase over baseline value. Concentration-response curves of the BDL/STZ/K group were significantly weaker than that of the BDL/K and BDL/G groups (mean±SDM). (B) Concentration-response curves of BDL/STZ rats. K: Krebs solution; G: D-glucose; OPC-31260: OPC-31260, expressed as absolute increase over baseline value. Concentration-response curves of the BDL/STZ/OPC-31260 group were significantly higher in 10^−7^ M AVP than that of the BDL/STZ/K and BDL/STZ/G groups.

Figure 2A depicts the comparison between BDL and BDL/STZ rats perfused with Krebs solution or D-glucose (BDL/K, BDL/G, BDL/STZ/K and BDL/STZ/G). Compared to the BDL/K group, the perfusion pressure changes to AVP were significantly alleviated in BDL/STZ/K group (10 µM: 10.4±2.22 vs. 6.71±2.43, p = 0.018). Similar result was also observed between the BDL/G and BDL/STZ/G groups (30 nM: 12.78±2.91 vs. 8.89±1.83, *P* = 0.033). There was no significant difference between the BDL/K and BDL/G or the BDL/STZ/K and BDL/STZ/G groups.

#### b. STZ-injected BDL rats with Krebs solution, D-glucose, or OPC-31260 perfusion

Figure 2B reveals the concentration-response curves of BDL/STZ rats perfused with Krebs solution, D-glucose, or OPC-31260 (BDL/STZ/K, BDL/STZ/G and BDL/STZ/OPC-31260). The perfusion pressure change of the BDL/STZ/OPC-31260 group was significantly higher than that of the BDL/STZ/K (10 µM: 11.88±3.64 vs. 6.71±2.43, p = 0.004) and BDL/STZ/G (10 µM: 11.88±3.64 vs. 7.44±1.88, *P* = 0.009) groups.

#### c. Maximum increases of perfusion pressure correlated with the blood glucose concentrations of vehicle- and STZ-injected BDL rats

**Figure 3:**
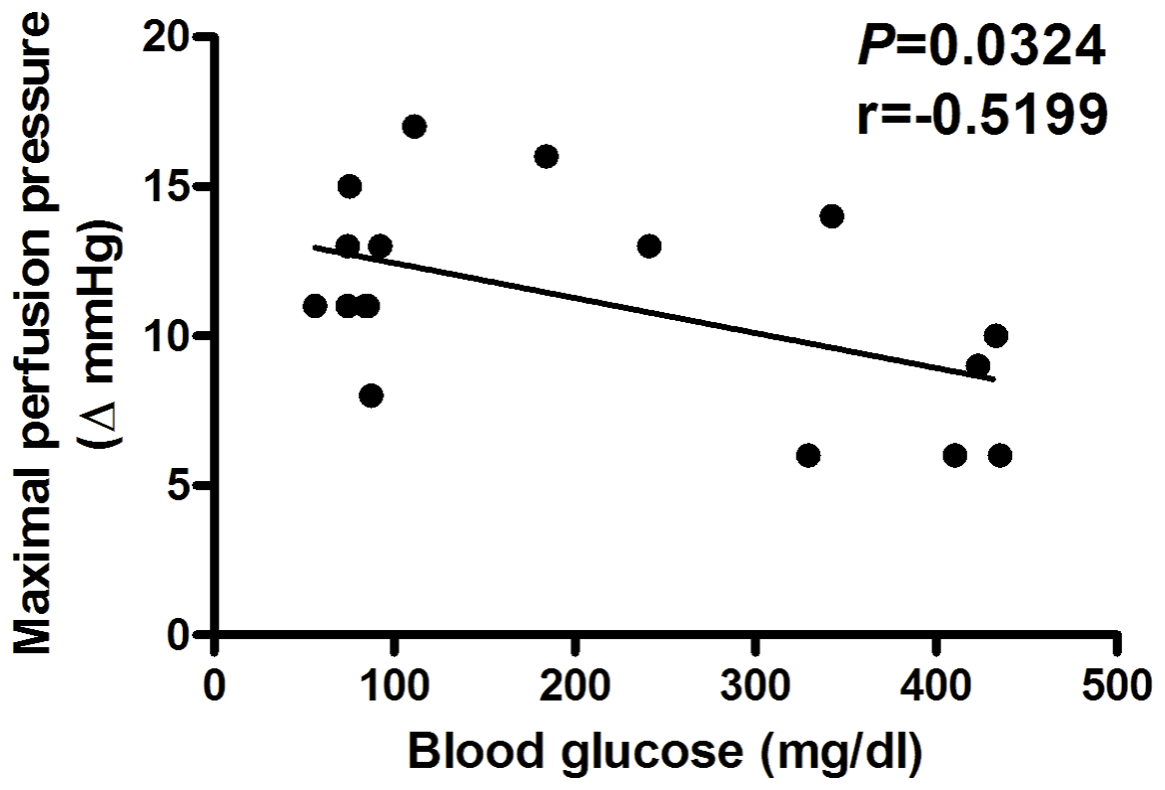
Correlations between maximal increases of perfusion pressure and the blood glucose concentrations in vehicle- and STZ-injected BDL rats with Krebs solution perfusion. The maximal perfusion pressure changes to AVP were negatively correlated with their blood glucose concentrations in the BDL/K and BDL/STZ/K groups (*P* = 0.0324, r = 0.5199).

Figure 3 demonstrated that the maximal perfusion pressure changes of the BDL/K and BDL/STZ/K groups negatively correlated with their blood glucose concentrations (*P* = 0.0324, r = −0.5199).

#### d. Maximum increase of perfusion pressure and EC50 to AVP of STZ-injected BDL rats with different pre-incubations

Table 3 shows the maximum increase of collateral perfusion pressure and EC50 of the BDL/STZ rats with Krebs solution, D-glucose, or D-glucose and NaF perfusion (BDL/STZ/K, BDL/STZ/G, BDL/STZ/G+NaF). The maximum perfusion pressure changes to AVP of the BDL/STZ/G+NaF group were significantly higher than those of the BDL/STZ/K and BDL/STZ/G groups (*P*<0.001). The EC50 of BDL/STZ/G+NaF group was also significantly lower than those of the BDL/STZ/K and BDL/STZ/G groups (*P*<0.001). The maximum perfusion pressure and EC50 were also evaluated in BDL rats perfused with D-glucose and NaF (BDL/G+NaF). There were no significant differences between BDL/G+NaF and BDL/STZ/G+NaF groups (*P*>0.05).

**Table 3:** Maximum increase of perfusion pressure and EC50 in BDL rats with STZ injection.

	n	Maximum increase (mmHg)	EC50 (nM)
BDL/G+NaF	7	19.71±2.98	2.023±0.370
BDL/STZ/K	7	9.14±3.39†*	3.005±0.336†*
BDL/STZ/G	9	9.67±2.65†*	2.973±0.336†*
BDL/STZ/G+NaF	6	17.5±4.55	1.975±0.458

† : *P*<0.01 vs. BDL/STZ/G+NaF rat; *: *P*<0.01 vs. BDL/G+NaF rat.

#### e. Splenorenal shunt vasopressin receptors mRNA expressions

In figure 3, splenorenal shunt mRNA expression of V_1a_R was not different between BDL and BDL/STZ groups (V_1a_R/β-actin: 0.001673±0.000348 vs. 0.00205±0.000708, *P*>0.05). However, the BDL/STZ group had a higher V_2_R mRNA expression than that of the BDL group (V_2_R/β-actin: 0.000037±0.000020 vs. 0.000152±0.000100, *P* = 0.023).

#### f. Splenorenal shunt G_α_ family proteins mRNA expressions and correlations with the maximal AVP responsiveness

**Figure 4:**
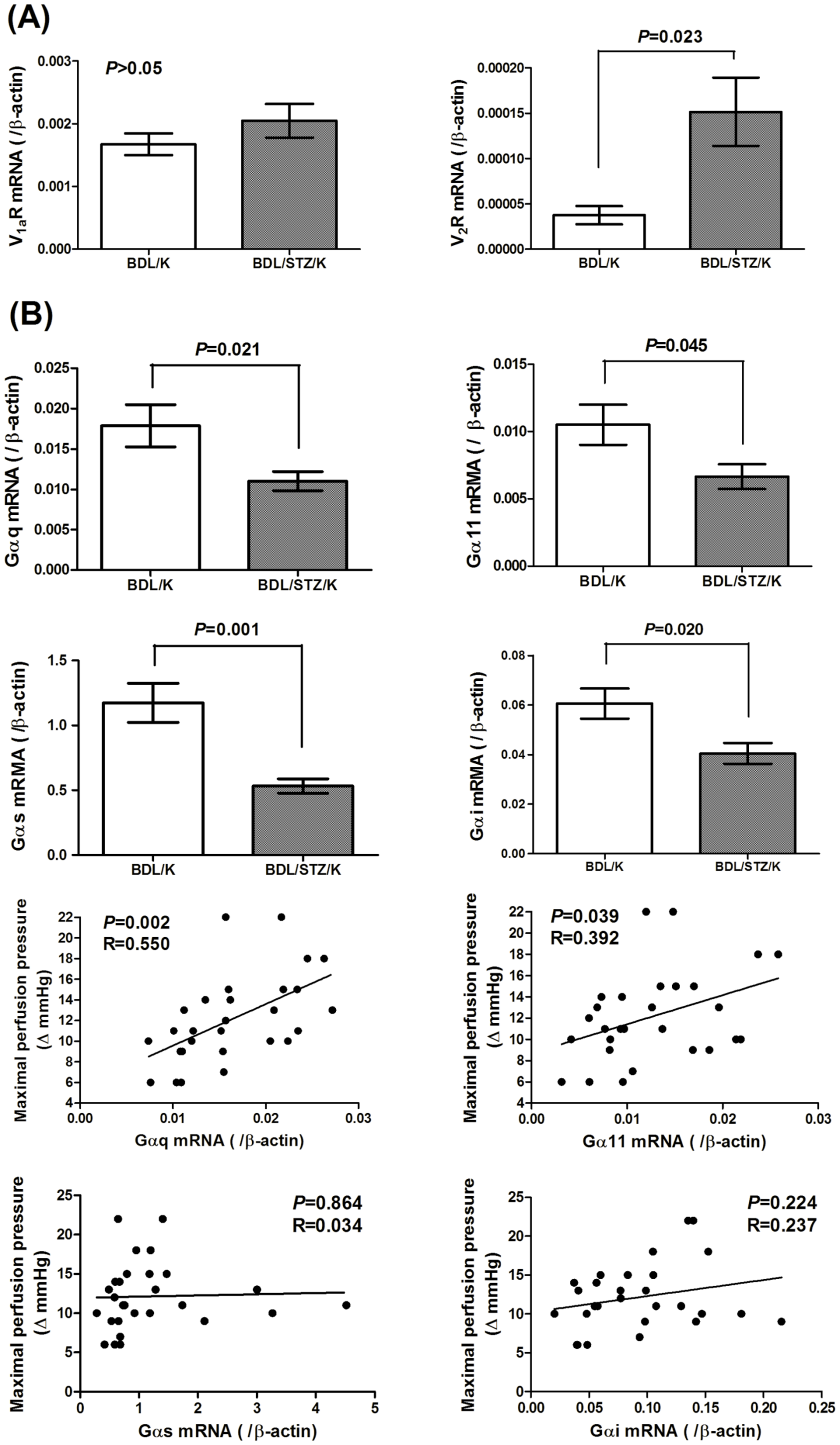
V_1a_R, V_2_R and G_α_-protein mRNA expressions in splenorenal shunts of BDL or BDL/STZ rats. (A) BDL: vehicle injection; BDL/STZ: STZ injection; K: Krebs solution. V_1a_R mRNA expression level was not significantly different between BDL/K and BDL/STZ/K groups. V_2_R mRNA expression was significantly lower of the BDL/K group than that of the BDL/STZ/K group. (B) G_α_-protein expressions and their relationship with the maximal AVP responsiveness. BDL: vehicle injection; BDL/STZ: STZ injection; K: Krebs solution. Compared to BDL/K group, G_αq_, G_α11_, G_αs_ and G_αi_ mRNA expression levels were all significantly decreased in the BDL/STZ/K group (*P*<0.05). The maximal perfusion pressure change to AVP was positively correlated with G_αq_ (*P* = 0.002, R = 0.550) and G_α11_ expression (*P* = 0.039, R = 0.392).

Figure 4 reveals the splenorenal shunt mRNA expressions of G_αq_, G_α11_, G_αs_ and G_αi_ proteins. Compared to the BDL group, the BDL/STZ group had significantly decreased mRNA expressions of G_αq_, G_α11_, G_αs_ and G_αi_ proteins (G_αq_/β-actin: 0.01788±0.00520 vs. 0.01102±0.00312, *P* = 0.021; G_α11_/β-actin: 0.01051±0.00297 vs. 0.00666±0.00244, *P* = 0.045; G_αs_/β-actin: 1.174±0.302 vs. 0.533±0.146, *P* = 0.001; G_αi_/β-actin: 0.0606±0.0122 vs. 0.0404±0.0111, *P* = 0.020).

Simple linear regression analysis between the G_αq_, G_α11_, G_αs_ and G_αi_ mRNA expressions and the maximal perfusion pressure changes in the BDL and BDL/STZ groups showed a significant correlation of G_αq_ and G_α11_ expressions with the maximal perfusion pressure changes (*P* = 0.002, R = 0.550 and *P* = 0.039, R = 0.392, respectively). However, G_αs_ and G_αi_ expressions did not correlate with the maximal perfusion pressure changes (*P*>0.05).

## Discussion

The present study indicates that diabetes diminishes the portal-systemic collateral AVP response in cirrhotic rats, which may be related to V_2_ receptor up-regulation and G_α_ proteins down-regulation. This is the first time that an animal model with BDL-induced cirrhosis and STZ-induced diabetes is established.

In this study, the collateral AVP vasoresponse was poorer in hyperglycemic than in normoglycemic BDL rats. Furthermore, the maximal perfusion pressure changes of hyperglycemic and normoglycemic BDL rats negatively correlated with their blood glucose concentrations. Previous studies also demonstrated that the decreased vasocontractile response in diabetic animals [22], [23] and the impaired AVP response may be due to vasopressin receptors desensitization [24]. Nevertheless, regional differences and experimental settings should be considered, since AVP response is reduced in coronary arteries but increased in renal arteries in STZ-diabetic rats [25].

Acute glucose exposure is deleterious for endothelial cells and vasodilatory action in diabetic and normal humans [26]. Impaired vasorelaxation has also been described in diabetic rats [14]. However, out data showed that D-glucose perfusion did not modify AVP responsiveness in cirrhotic rats with or without STZ-induced diabetes. This implies that acute glucose administration poses a less prominent influence on collateral vasoresponse than chronic hyperglycemia.

Vasopressins act through G protein-coupled receptors V_1a_R, V_1b_R, and V_2_R. V_1_R down-regulation alleviates AVP response in diabetes [27], [28]. However, splenorenal shunt V_1a_R mRNA expression was not different between BDL/K and BDL/STZ/K groups, indicating that the alleviated collateral AVP response in hyperglycemic BDL rats was not related to V_1a_R. Surprisingly, V_2_R mRNA expression was enhanced in BDL/STZ/K group and the V_2_R antagonist enhanced the collateral AVP response, suggesting that the collateral hyporesponsiveness of the diabetic cirrhotic rat is at least partly ascribed to V_2_R up-regulation. Consistently, V_2_R mediated vasorelaxation to AVP in diabetic patients and animals [29]–[31].

The splenorenal shunt G_αq_, G_α11_, G_αs_ and G_αi_ mRNA expressions were down-regulated in STZ-injected BDL rats. Although G_αs_ mediates vasorelaxation and G_αq_, G_α11_, G_αi_ elicit vasoconstriction, G_αs_ down-regulation did not overcome the net effect of G_αq_, G_α11_ and G_αi_ down-regulations. Furthermore, the collateral maximal pressure changes correlated with G_αq_ and G_α11_ expressions, but not G_αi_ and G_αs_. Preincubation with NaF, a G protein activator in STZ-injected cirrhotic rats increased the maximal pressure changes and decreased the EC50 to AVP as compared with those without. NaF also abrogated the difference of maximal AVP response between BDL and BDL/STZ rats. Taken together, an ameliorated collateral AVP responsiveness of cirrhotic rats with diabetes is, at least partly, associated with G_α_ down-regulation.

In the current study, cirrhotic rats with diabetes had significantly lower BW than those without diabetes. Decreases in BW, MAP and HR had been found in STZ-diabetic rats [32]. One concern is if the difference in BW influences vascular tone. The lower weight may be caused by dehydration related to diabetes and/or decreased adipose tissue. Adipose tissue elevates estrogen levels that cause nitric oxide (NO)-mediated vasodilatation [33], [34], while dehydration stimulates vasoconstriction via release of endogenous vasopressin [35]. Accordingly, the collateral vasocontractile response could be enhanced in STZ-injected BDL rats. However, the present study demonstrates the opposite. It can be inferred that BW loss does not affect collateral vasoresponse in cirrhotic rats with diabetes.

This study has some limitations. The perfusion experiments could only be performed on the survived. Therefore, the vasoresponse between survivors and non-survivors could not be compared. Furthermore, the perfusion study could not be performed on sham-operative rats since they do not develop the pathological portal-systemic collaterals. Another concern is the potential influences of different animal models on the vasoresponsiveness. BDL is a well-established technique to induce secondary biliary cirrhosis with NO over-expression and hepatopulmonary syndrome [36]. It has been applied in our previous studies addressing the vasoresponsiveness in cirrhosis [37], [38]. Hepatotoxins, such as carbon tetrachloride (CCl_4_), have also been used to elicit cirrhosis. However, BDL could be more feasible for vascular study since CCl_4_ induced vascular endothelial damage in both animal and human studies [39], [40], which might adversely influence the finding.

The survival rate of STZ-injected BDL rats (49.3%) was significantly lower than that of vehicle-injected BDL rats (87.1%). Consistently, the five-year cumulative survival rate of cirrhotic patients with normal glucose tolerance was 94.7%, whereas that of cirrhotic patients with diabetes was 56.6% [41]. In another study, diabetes exacerbated liver failure and increased mortality in cirrhotic patients [42]. Our data are quite reliable since the experiments have been done on animals of the same species, raised in the same highly controlled environment, and fully randomized.

In conclusion, cirrhotic rats with diabetes exert poorer portal-systemic collateral AVP response than normoglycemic cirrhotic rats. This is related to vascular V_2_R up-regulation and G proteins down-regulation, especially G_αq_ and G_α11_. Acute glucose perfusion does not influence collateral AVP responsiveness, suggesting that long-term glycemic control is pivotal for improving the collateral vasocontractile response during variceal bleeding in cirrhosis. Furthermore, G_α_ protein signaling pathway may be a potential therapeutic target.
